# Epidemiological and evolutionary management of plant resistance: optimizing the deployment of cultivar mixtures in time and space in agricultural landscapes

**DOI:** 10.1111/eva.12304

**Published:** 2015-10-23

**Authors:** Frédéric Fabre, Elsa Rousseau, Ludovic Mailleret, Benoît Moury

**Affiliations:** 1 UMR 1065 Unité Santé et Agroécologie du Vignoble, INRAVillenave d’Ornon Cedex, France; 2 Biocore Team, INRIASophia Antipolis, France; 3 UMR 1355 Institut Sophia Agrobiotech, INRASophia Antipolis, France; 4 UMR 7254 Institut Sophia Agrobiotech, Université Nice Sophia AntipolisSophia Antipolis, France; 5 UMR 7254 Institut Sophia Agrobiotech, CNRSSophia Antipolis, France; 6 UR 407 Pathologie Végétale, INRAMontfavet, France

**Keywords:** evolutionary epidemiology, functional connectivity, heterogeneity of selection, landscape epidemiology, mosaic strategy, qualitative resistance, resistance durability, rotation strategy

## Abstract

The management of genes conferring resistance to plant–pathogens should make it possible to control epidemics (epidemiological perspective) and preserve resistance durability (evolutionary perspective). Resistant and susceptible cultivars must be strategically associated according to the principles of cultivar mixture (within a season) and rotation (between seasons). We explored these questions by modeling the evolutionary and epidemiological processes shaping the dynamics of a pathogen population in a landscape composed of a seasonal cultivated compartment and a reservoir compartment hosting pathogen year-round. Optimal deployment strategies depended mostly on the molecular basis of plant–pathogen interactions and on the agro-ecological context before resistance deployment, particularly epidemic intensity and landscape connectivity. Mixtures were much more efficient in landscapes in which between-field infections and infections originating from the reservoir were more prevalent than within-field infections. Resistance genes requiring two mutations of the pathogen avirulence gene to be broken down, rather than one, were particularly useful when infections from the reservoir predominated. Combining mixture and rotation principles were better than the use of the same mixture each season as (i) they controlled epidemics more effectively in situations in which within-field infections or infections from the reservoir were frequent and (ii) they fulfilled the epidemiological and evolutionary perspectives.

## Introduction

Integrating the principles governing natural ecosystems into crop protection strategies should be a powerful way to face the challenge of doubling crop production in the next four decades while decreasing the environmental impact of agriculture (Tilman 1999). In natural ecosystems, the genetic diversity of hosts limits the spread of epidemics in a wide range of conditions (Ostfeld and Keesing 2012). In agroecosystems, functional diversity in disease resistance also decreases disease spread (Mundt 2002; Garrett et al. 2009). This approach has been particularly successful in the control of powdery mildew in barley and blast disease in rice (Wolfe 1985; Zhu et al. 2000). Limited efficacy has also been often reported (e.g., Garrett et al. 2009).

Disease resistance in plants often results from a molecular relationship governed by a gene-for-gene interaction (Flor 1971). For qualitative resistance genes (R genes) (i.e. genes that almost totally prevent plant infection), the interaction between the product of the R gene of the plant (which has at least two allelic forms: ‘resistant’ and ‘susceptible’) and the product of the avirulence gene of the pathogen (which has at least two allelic forms: ‘nonadapted’ and ‘resistance-breaking’) determines the resistance or susceptibility of the plant. Over the last decade, the molecular dissection of these interactions for viruses has revealed that (i) one or two nucleotide substitutions in virus avirulence genes are often sufficient to overcome resistance genes (Jenner et al. 2002; Kang et al. 2005; Fraile et al. 2011; Moury et al. 2011) and (ii) these substitutions have variable fitness costs in susceptible plants (Ayme et al. 2006; Janzac et al. 2010).

Pathogen adaptation is a key factor limiting the usefulness of mixtures of susceptible and resistant cultivars. In natural ecosystems, R genes may remain effective for long periods, because stable polymorphism is maintained by mechanisms such as negative frequency-dependent selection (Lewontin 1958; Brown and Tellier 2011; Zhan et al. 2015). Most of these mechanisms have been lost in modern agroecosystems. For example, cultivar selection by growers disrupts plant–pathogen coevolution (Bousset and Chèvre 2013). R genes thus often remain effective for only short periods, particularly for fungi and bacteria (McDonald and Linde 2002), but also for viruses (García-Arenal and McDonald 2003). This results in a classic ‘boom-and-bust’ cycle, in which cultivars carrying a new resistance gene are widely adopted by farmers, leading to a breakdown of resistance and replaced with new cultivars carrying another R gene. This cyclic system works well if sufficient R genes are available in the genetic resources for the plant species concerned. Unfortunately, R genes, particularly qualitative ones, are rare resources requiring careful, sustainable management.

The interplay between the spatial scale over which an epidemic spreads and that at which host heterogeneity is distributed also limits the usefulness of cultivar mixtures. One key reason for this is the need for control strategies to be applied at the same spatial scale as that of epidemic spread (Dybiec et al. 2004; Gilligan et al. 2007; Gilligan 2008). As many airborne and vectorborne diseases spread over long distance, epidemiologists and agriculture managers are increasingly focusing on the spatial scale of the landscape (Plantegenest et al. 2007; Real and Biek 2007; Parnell et al. 2009, 2010; Skelsey et al. 2010; Papaïx et al. 2011). At this scale, epidemics in individual fields follow two routes of infection, originating within the same field or in other fields (Park et al. 2001). Their relative importance depends on the spatial grain of the distribution of host heterogeneity in the landscape. This spatial grain is a component of landscape connectivity, ‘the degree to which the landscape facilitates or impedes movement among resource patches’ (Taylor et al. 1993). It remains unclear how landscape connectivity influences the interaction between uninfected and infected individuals (Meentemeyer et al. 2012), thereby affecting epidemics. The role of landscape connectivity in pathogen evolution must also be considered in the management of resistance sustainability at the landscape scale (Papaïx et al. 2013; Mundt 2014).

We used a model coupling epidemiology and population genetics to study how mixtures of susceptible and resistant cultivars can control epidemics efficiently, over several seasons at the landscape scale, while preventing the emergence of adapted pathogens. Specifically, two management alternatives were considered (Zhan et al. 2015). In the first, the objective is to maximize crop yield (yield strategies). In the second, there are two objectives: to decrease yield loss due to pathogens and to slow the evolution of adapted pathogens (sustainable strategies). We investigated the role of landscape features (connectivity and epidemic intensity before the introduction of the resistant cultivar) and that of the molecular interactions between plant and pathogen. We found that the knowledge of the interaction between these factors is required to strategically associate susceptible and resistant cultivars in mixture and rotation.

## Materials and methods

### Model overview

The model couples plant epidemics and pathogen population genetics processes during a succession of cropping seasons. First, it describes during a cropping season the dynamics of epidemics in a landscape composed of susceptible (S) and resistant (R) plants and changes in the frequency of a resistance-breaking variant in the pathogen population. It deals with the case of any plant–pathogen for which the within- and between-host dynamics are clearly separated, typically a virus. Second, epidemics in successive cropping seasons are coupled to one another through the interaction of the crop with a reservoir compartment, containing diverse wild plant species that may serve as hosts for the pathogen during the crop-free period and as a source of inoculum for the initiation of infections in the next cropping period. In this landscape, we can consider three routes of infection: (i) between the reservoir and the fields, (ii) between fields, and (iii) within a field. The main variables and parameters of the model are listed in Table1.

**Table 1:** Description of the parameters of the model of their range of variation and of the state variables of the model

Parameters	Designation (Reference value)	Unit	Sensitivity analyses levels
Ω_int_	Epidemic intensity before R deployment (in a landscape with only S plants)*,‡	Unitless	4 levels: 0.1, 0.3, 0.5, 0.8
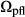	Landscape connectivity before R deployment (in a landscape with only S plants)†,‡	Unitless (vector)	4 levels: 1 (0.05, 0.05, 0.9), 2 (0.05, 0.9, 0.05), 3 (0.9, 0.05, 0.05), 4 (1/3, 1/3, 1/3)
*λ*	Characteristic of the pathogen reservoir§ (0.5)	Unitless	3 levels: 0.1, 0.5, 0.9
*θ*	Choice of R gene (defining the frequency of the resistance-breaking variant in S plants)¶	Unitless	5 levels: 10^−8^, 10^−6^, 10^−4^, 10^−2^, 0.5
*n*_*y*_	Number of years of resistance deployment (15)	Year	2 levels: 10, 20
*n*_d_	Duration of the cropping season (120)	Day	
*n*_f_	Number of fields in the landscape (400)	Field	
*n*_p_	Number of plants in a field (10^4^)	Plant	

State variables	Designation	
*I*_S,*y*_	Number of infected plants in a field with the S cultivar during year *y*	Plant
*I*_R,*y*_	Number of infected plants in a field with the R cultivar during year *y*	Plant
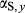	Rate of infection of the S cultivar from the reservoir during year *y*	Day^−1^
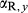	Rate of infection of the R cultivar from the reservoir during year *y*	Day^−1^

* In a landscape with only the susceptible cultivar, the epidemic intensity is the mean frequency of S plants infected in a field during a cropping season.

† The parameters 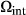 and 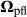 define the epidemic dynamics in the landscape before the deployment of the resistant cultivar where epidemics repeat themselves identically every year.

‡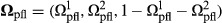. In a landscape with only the susceptible cultivar, 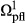 measures the frequency of infection events originating from the reservoir, 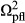 the frequency of between-field infection events and 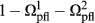 the frequency of within-field infection events. 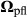 defines the connectivity between the elements (fields, reservoir) of the landscape.

§ *λ* represents the degree of decrease in the weighting of the reservoir pathogen load in an exponential moving average setting. Higher *λ* values result in the faster discounting of older reservoir pathogen loads. The levels of *λ* account for a wide range of the possible reservoir, with pathogen populations having a half-life of ≈6 months (*λ = *0.9), ≈1 year (*λ = *0.5) and ≈ 6 years (*λ = *0.1).

¶ The choice of qualitative R gene by plant breeders determines the number and fitness costs of the nucleotide substitutions that nonadapted pathogens must accumulate in their avirulence gene to overcome the R gene. In turn, these parameters determine the frequency of coexistence of resistance-breaking and nonadapted variants in S plants. The levels of *θ* account for resistance genes requiring 1 or 2 nucleotide substitutions in the avirulence gene of the pathogen to be broken down. The case of an RNA plant virus is addressed more specifically here. When only one (resp. two) mutation is required, assuming a mutation rate of 10^−4^ and given the distribution of fitness effects of single mutations (Carrasco et al. 2007), *θ* is likely to be in the range [10^−4^, 0.01] (resp. [10^−8^, 10^−6^]). *θ* = 0.5 represents a situation in which one mutation with a very low (2 × 10^−4^) fitness cost is required.

### Within-season model of a landscape with only S fields

As a first step, the model was described verbally for a landscape with several fields in which only the S cultivar was sown (‘S fields’). This situation also defines the baseline epidemiological situation before the deployment of the R plants. The variable modeled was *I*_S,*y*_(*t*), the number of plants infected in a S field at time *t* during year *y*. Its dynamic is governed by two processes: (i) the mean epidemic intensity in a field during a season (parameter 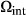) and (ii) the relative importance of the three routes of infection (vector of parameter 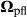). The vector 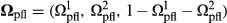 captures the infection profile of the landscape, which is dependent on the connectivity between its elements (fields, reservoir). More specifically, 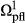 is the proportion of infections originating from the reservoir, 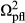 is the proportion of between-field infections, and 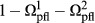 is the proportion of within-field infections. These parameters are an intuitive and powerful way to characterize agricultural landscapes from an epidemiological point of view before resistance deployment (‘R deployment’) (Fabre et al. 2012a).

With only S fields, before R deployment, epidemics *I*_S,*y*_(*t*) repeat themselves identically from year to year during *n*_*y*_ successive cropping seasons. The virus load of the reservoir, which is proportional to *α*_S,*y*_, the rate of infection of the S cultivar from the reservoir during year *y*, also remains constant between years.

### Within-season model of a landscape with S and R fields

The inclusion of a R cultivar leads to additionally consider the variable *I*_R,*y*_(*t*) of the number of plants infected in a field sown with the R cultivar (‘R fields’) at time *t* during year *y*. We considered a qualitative resistance. We therefore defined two pathogen variants: the nonadapted and the resistance-breaking variants, interacting in a gene-for-gene manner. The R cultivar can only be infected by the resistance-breaking variant. The S cultivar can be infected by both variants. We assumed that, on S plants, the nonadapted and resistance-breaking variants coexist in a mutation-selection balance. This equilibrium, characterized by parameter *θ*, results from the balance between the production of resistance-breaking variants through recurrent mutations of nonadapted variants and their counter-selection because of the fitness costs associated with the adaptive mutations. For a given mutation rate, *θ* depends on (i) the number of nucleotide substitutions required for the pathogen to break down the resistance conferred by the R gene and (ii) their impact on the competitiveness of the virus. *θ* can also be seen as characteristics of the R gene selected by breeders.

### Between-season model in a landscape with both S and R fields

As soon as R fields occupy a proportion *φ*_*y*_ of the landscape during year *y*, the epidemic dynamics are no more of intensity Ω_int_ and profile **Ω**_pfl_: they change from season to season. In particular, the rates *α*_S,*y*_ and *α*_R,*y*_, the rate of infection of the R cultivar from the reservoir during year *y*, will change each year, as a function of (i) the overall intensity of the epidemic in the R and the S fields in the previous year, as lower epidemic intensities decrease the pathogen load of the reservoir and (ii) the lifespan of the plants of the reservoir and the relative sizes of the cultivated and reservoir compartments. These two characteristics are summarized by a single parameter *λ* controlling the rate of renewal of the pathogen load in the reservoir, with high values of *λ* characteristic of a reservoir that is rapidly changing due to short host life spans and small reservoir size. Importantly, it is assumed that the plants of the reservoir are selectively neutral for the pathogen population. Thus, between seasons, the reservoir conserves the relative frequencies of the nonadapted and resistance-breaking variants arising from the crops. It is assumed that the frequency of the resistance-breaking variant in the reservoir is *θ* before the introduction of the R cultivar in the landscape.

### Mathematical description of the model

We provide here a formal mathematical description of the model. The model is semidiscrete (Mailleret and Lemesle 2009). It mostly follows continuous dynamics described by ordinary differential equations (ODE), but, at given time points, it displays discrete dynamics. Thereafter, *n*_*y*_ cropping seasons are considered, each lasting *n*_d_ days. The landscape consists of *n*_f_ fields, with a proportion *φ*_*y*_ of R fields during year *y*, with each field containing *n*_p_ plants.

The ODEs describing epidemics during cropping season *y* are



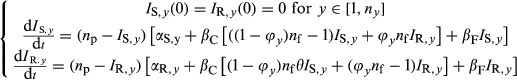
1

Assuming that farmers sow healthy plants, the infections of S and R plants are initiated from the reservoir at rates *α*_S,*y*_ and *α*_R,*y*_, respectively. S plants may also be infected by pathogens from plants growing in the same field (rate *β*_F_) or by pathogens from infected plants growing in other fields (rate *β*_C_), regardless of the type of cultivar concerned. The same processes govern the infection of R plants, except that these plants can only be infected by pathogens from fields of S plants, at a rate discounted by *θ*.

The discrete equations describing the interseason dynamics of the pathogen load of the reservoir are based on an exponential moving average of weight *λ*:

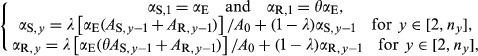
2where 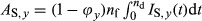, 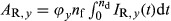 and 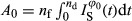 [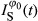 is defined below]. Thus, the lower the intensity of the overall epidemic in both R and S fields during year *y *− 1 (i.e. 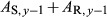) relative to the overall epidemic intensity of a landscape with only S fields (i.e. *A*_0_), the larger the decrease in the pathogen load of the reservoir from season *y* − 1 to *y*. Similarly, higher values of *λ* correspond to a faster drop-off of pathogen levels from older epidemics.

Before R deployment, with only S fields, 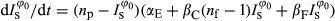 with 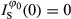. In this situation, the rates 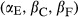 defining the intensities of the three routes of infection are easier to interpret by considering the alternative parameters 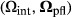 (Table S1, Fabre et al. 2012a).

Transformation techniques aiming to put the model in a dimensionless form showed that the results presented were independent of *n*_p_ and *n*_f_, for high values of *n*_f_, which is a reasonable assumption for agricultural landscapes. Moreover, using 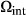 and 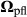 to define epidemics ensured that the results were independent of season length *n*_d_. Epidemics characterized by 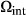 and 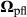 can occur over seasons of different lengths, provided that the epidemic parameters *α*_E_, *β*_F_, and *β*_C_ are rescaled appropriately.

### Model analysis

#### Measuring the yield increase achieved with deployment strategies

A deployment strategy 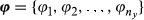 is the time series of the proportions of fields sown with R plants each year in the landscape during the time window of resistance deployment considered (*n*_*y*_). For year *y*, the sum of the area under the disease progress curves *I*_S,*y*_(*t*) and *I*_R,*y*_(*t*) weighted by the proportions 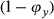 of S fields and *φ*_*y*_ of R fields is a proxy for yield losses due to the pathogen. Formally, these quantities are the integrals *A*_S,*y*_ and *A*_R,*y*_ defined above. The overall yield loss over *n*_*y*_ years is given by 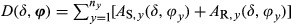, where 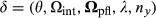 is a given set of model parameters.

The performance of the deployment strategy ***φ*** is measured by the relative damage 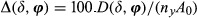. Dividing 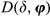 by *n*_*y*_*A*_0_, the overall yield losses obtained in a landscape containing only S fields, provides an estimate of the damage obtained with ***φ*** relatively to the damage that would have been obtained without using the R cultivar. For example, a value of 80% indicates that the strategy ***φ*** decreases damage due to the pathogen by 20%.

### Performance of R deployment strategies maximizing yield

The model was analyzed to explore how the yield increase obtained with optimal strategies of R deployment depended on five parameters of interest 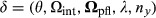. We first considered strategies in which the same proportion of R fields was sown every year. These so-called constant-mixture yield strategies (CYS) are designed by determining the proportion 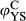 of R fields minimizing 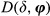.

We then determined to what extent the performance of CYS [measured by the relative damage 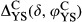] could be improved by modifying the proportion of R fields every 3 years. The resulting so-called variable-mixture yield strategies (VYS) were designed, for *n*_*y*_ = 15 only, by determining five proportions of R fields 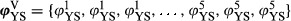 minimizing 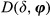, each proportion being applied during three successive cropping seasons. The performance of VYS was measured (i) as described above, by determining the relative damage 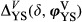 and (ii) by determining the additional relative benefit of these strategies 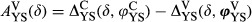, corresponding to the percentage difference in damage between the VYS and the reference CYS. A value of 10% indicates that the VYS is 10% points more beneficial than the CYS.

### Performance of R deployment strategies maximizing both yield and resistance durability

We then focused on sustainable strategies designed to maximize yield while remaining sustainable. CYS and VYS strategies with the sole objective of maximizing yield are not necessarily sustainable. There is no reason that the frequency of the resistance-breaking variant will remain low.

We again first considered sustainable strategies in which the same proportion of R fields was sown in each year. These so-called constant-mixture sustainable strategies (CSS) were designed by determining the proportion 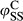 of R fields minimizing 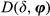, while ensuring that the mean proportion of infected R plants remained below the threshold value of 5% for all R fields in all years. We analyzed the difference in yield performance between CSS [measured by the relative damage 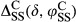] and CYS.

We then carried out the same analysis with variable-mixture sustainable strategies (VSS), defined by determining the five proportions of R fields 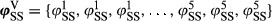 minimizing 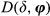 while ensuring that the mean proportion of infected R plants remained below 5%, for all fields and all years. These strategies were identified only for *n*_*y*_ = 15, each proportion being applied during three successive cropping seasons. We analyzed the difference in yield performance between VSS [measured by the relative damage 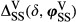] and CYS.

### Implementation of model analyses

The model and analyses were implemented in R software (http://www.r-project.org/). The model was solved with the ‘lsoda’ function (library ‘deSolve’). Optimal strategies were identified with a Nelder–Mead algorithm (detailed in Appendix S1 and Figure S1). The numerical exploration of the model was performed by combining global sensitivity analysis, one-at-a-time analysis and hierarchical clustering methods (Appendix S2 and Table S1).

## Results

### Analysis of deployment strategies maximizing yield

#### Sensitivity analyses

Sensitivity analyses of the relative damage obtained with constant-mixture yield strategies (CYS) indicated that three of the five parameters of interest considered were important (Figure S2A) given the large range of production situations explored (Table1). The sum of the main indices (revealing the individual effect of factors) of epidemic intensity, landscape connectivity and choice of R gene accounted for 82% of the variance for relative damage. The variance explained rose to 95% when we added their second- and third-order interactions. The most influential factors were the intensity of the epidemic before the deployment of R plants (accounting for 42% of the variance), followed by the choice of R gene (31%). The third factor, landscape connectivity, accounted for almost 10% of the variance. By contrast, the characteristics of the reservoir and the duration of resistance deployment had only a marginal impact on the performance of the strategy.

#### Effect of the main factors

The effects of the choice of R gene and epidemic intensity were studied jointly for three contrasting patterns of landscape connectivity. Below, the range of the parameter *θ* is designated by a binary alternative. This parameter takes into account the number of mutations of the avirulence gene of the pathogen required to break down the resistance mediated by the R gene, and the fitness costs of these mutations. Given the probability distribution of the nonlethal fitness effects of single mutations in plant viruses (Carrasco et al. 2007), values of *θ* in the range [10^−4^, 0.01] are indicated a ‘R gene *typically* requiring 1 mutation to be broken down’ while values in the range [10^−8^, 10^−6^] indicated a ‘R gene *typically* requiring two mutations to be broken down’ (Table1, Fabre et al. 2012a).

**Figure 1:**
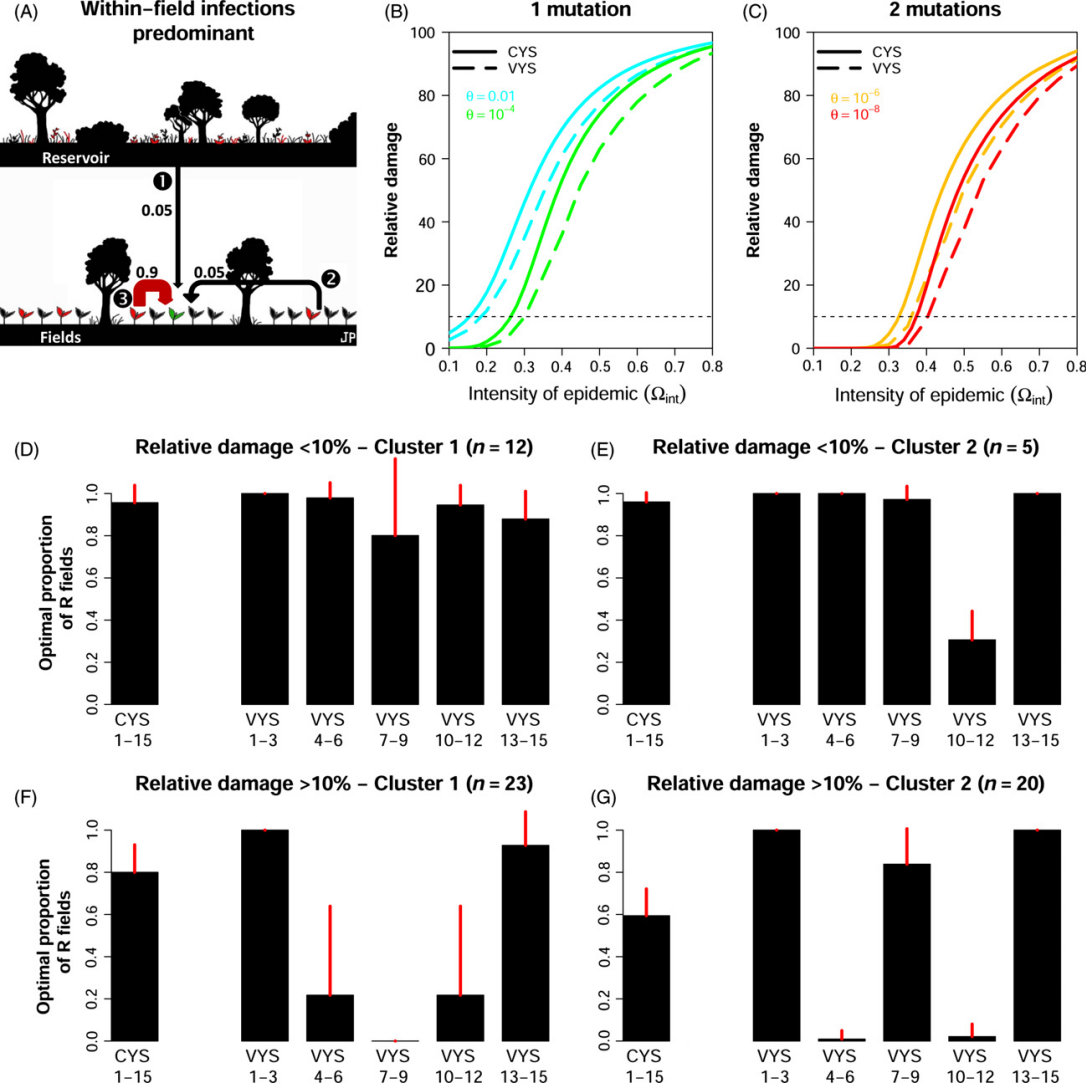
Comparison of the damage reduction achieved with constant-mixture and variable-mixture yield strategies in a landscape in which epidemic dynamics are driven mostly by within-field infections. (A) In this landscape, before the deployment of the resistant cultivar, 90% of the infections are within-field infections (arrow 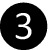). The other two infection routes, from the reservoir (arrow 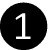) and between-field (arrow 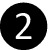), each accounts for 5% of the infection events. (B, C) The effects of resistant cultivar choice (*θ*) and of epidemic intensity before the deployment of the resistant cultivar (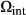) on the relative damage obtained with optimal constant-mixture yield strategies (CYS) and optimal variable-mixture yield strategies (VYS). Values of *θ* in the range [10^−4^, 0.01] (resp. [10^−8^, 10^−6^]) correspond to resistance genes typically requiring one (resp. two) mutation to be broken down, depending on the fitness costs associated with these mutations. The dotted line indicates relative damage of 10%, above this arbitrary threshold a substantial margin of improvement exists for CYS. (D, E). Clustering in two groups of the time series of the optimal proportion of R fields in VYS when the relative damage obtained with CYS is <10% (17 of the 60 parameter combinations displayed in graphs B and C). Bars show the mean (±standard deviation) of the proportion of R fields to sown in years 1–3, 4–6, 7–9, 10–12, and 13–15, and the proportion to sown with the CYS. (F, G) As for (D, E), but for relative damage obtained with CYS ≥10% (43 of 60 cases).

The case of a landscape in which epidemics are mostly driven by within-field infections is illustrated in Fig. 1. In this landscape, before the deployment of the resistant cultivar, 90% of the infections were within-field infections (Fig. 1A). This is the worst-case scenario for the control of epidemics with an R gene. CYS yield marked disease control only for the lower range of epidemic intensities. At best, damage is decreased by more than 90% only when epidemic intensities are <0.35 before the deployment of resistance (Fig. 1B,C). Choosing R genes harder to break down only slightly decreased the damage (Fig. 1C vs B).

In this landscape, the adoption of a variable-mixture yield strategy (VYS), in which the proportion of R fields is changed every 3 years, provided the greatest additional relative benefit. This was true regardless of the choice of R gene and, to a lesser extent, of epidemic intensity. When a margin of improvement exists for CYS (typically when their relative damages are >10%), the mean additional relative benefit was 8% and the highest values (up to 18%) were observed for intermediate epidemic intensities (Table S2). They were obtained only with highly periodic strategies, in which the optimal proportions of R fields varied from almost 1 to almost 0 (Fig. 1F,G). When CYS are already efficient (relative damage <10%), there is likely to be little gain with VYS. Alternation patterns for the optimal proportions of R fields are less contrasted (Fig. 1D,E).

**Figure 2:**
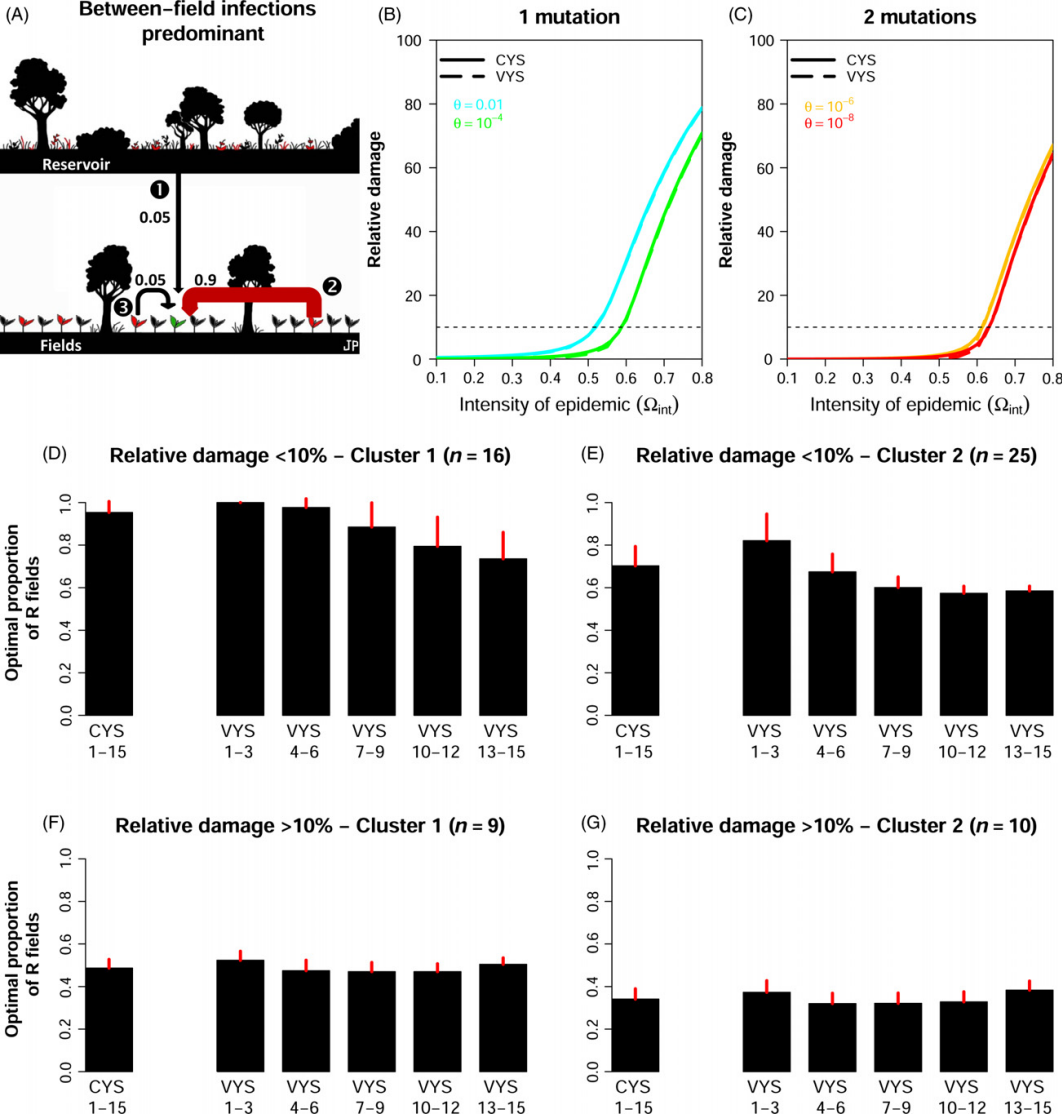
Comparison of damage reduction achieved with constant-mixture and variable-mixture yield strategies in a landscape in which epidemic dynamics are driven mostly by between-field infections. (A) In this landscape, before R deployment, 90% of infections are between-field infections (arrow 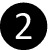). (B, C) Effects of the choice of resistant cultivar (*θ*) and of epidemic intensity before the deployment of the resistant cultivar (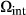) on the relative damage obtained with constant-mixture yield strategies (CYS) and variable-mixture yield strategies (VYS). (D, E). Clustering into two groups of the time series of the optimal proportion of R fields in VYS when the relative damage obtained with CYS is <10% (41 of the 60 parameter combinations displayed in graphs B and C). (F, G) As for (D, E), but for relative damage obtained with CYS ≥10% (19 of 60 cases). Please refer to Fig. 1 for further details.

The situation was very different for landscapes in which epidemics were driven mostly by between-field infections. In such landscapes, before the deployment of the resistant cultivar, 90% of infections are between-field infections (Fig. 2A). The range of epidemic intensities for which high levels of disease control (i.e., relative damage < 10%) were obtained with CYS was much larger and included all production situations in which epidemic intensity was below 0.5 before the deployment of the R cultivar (Fig. 2B), and even below 0.6 for resistance genes typically requiring two mutations to be broken down (Fig. 2C). However, for higher epidemic intensities, relative damage increased steeply, whatever the resistance gene.

Here, VYS were no more effective than CYS. The additional relative benefit accrued never exceeded 0.5% even when relative damages of CYS were > 10% (Table S2). The optimal proportions of R fields with VYS followed a smooth pattern (Fig. 2D–G). As for CYS, they were based on almost equal proportions of R and S fields, particularly for lower levels of disease control (i.e. relative damage > 10%) (Fig. 2F,G).

**Figure 3:**
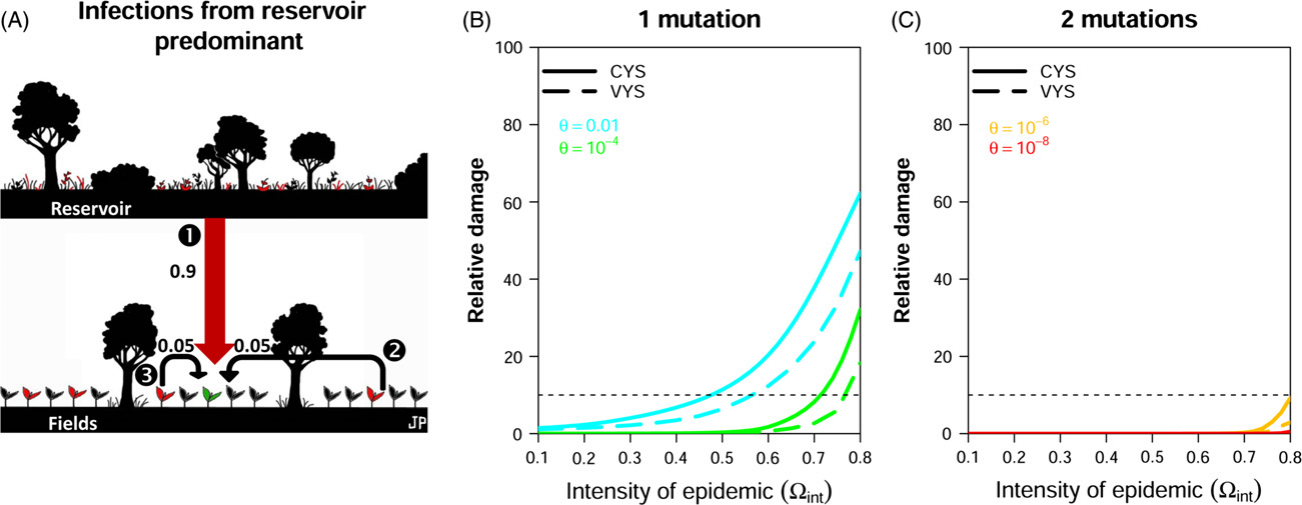
Comparison of damage reduction achieved with constant-mixture and variable-mixture yield strategies in a landscape in which epidemic dynamics are driven mostly by infections from the reservoir. (A) In this landscape, before R deployment, 90% of infections originate from the reservoir (arrow 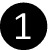). (B, C) Effects of the choice of resistant cultivar (*θ*) and of epidemic intensity before the deployment of the resistant cultivar (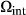) on the relative damage obtained with constant-mixture yield strategies (CYS) and variable-mixture yield strategies (VYS).

Finally, the case of a landscape in which epidemics are driven mostly by infections initiated from the reservoir is illustrated in Fig. 3. In such landscapes, before the deployment of the resistant cultivar, 90% of infections originate from the reservoir (Fig. 3A). Here, there is a substantial difference between R genes typically requiring only one mutation to be broken down (Fig. 3B) and R genes typically requiring two mutations to be broken down (Fig. 3C). When two mutations are required, high levels of disease control (i.e. relative damage < 10%) were achieved for the entire range of epidemic intensities explored. In most cases, pathogen damage was reduced by more than 99%, the optimal strategy being to use only the R cultivar. No additional benefit was obtained from the use of VYS. When a single mutation is typically required to break down the R gene (Fig. 3B), the performance of CYS depends on the fitness cost of the mutation. High fitness costs (*θ* = 10^−4^ in Fig. 3B) are associated with a high degree of disease control for epidemic intensities <0.7. This threshold falls to 0.45 for intermediate fitness costs (*θ* = 0.01 in Fig. 3B). For both fitness costs, VYS provided substantial additional benefit (mean of 11%; highest value 16%) when the relative damage of CYS was >10% (Table S2). These benefits were also obtained with highly periodic strategies (Figure S3, panels C and D).

The additional benefit obtained with VYS depended also of the rate of renewal of the pathogen load in the reservoir (Table S2). In slowly changing reservoir, they were always very low (<0.5%) in all landscapes (Table S2, *λ* *= *0.1). In rapidly changing reservoir, they were roughly doubled (from 8% to 17% for landscapes in which within-field infections predominated, from to 11% to 19% for landscapes in which infections from the reservoir predominated). Additional benefits still remained very low (<1%) for landscapes in which between-field infections predominated (Table S2, *λ *= 0.9).

### Analysis of deployment strategies maximizing yield while preserving resistance durability

Sensitivity analyses highlighted the same three factors as for CYS. Epidemic intensity, landscape connectivity, and choice of the R gene together accounted for 78% of the variance for the relative damage obtained with constant-mixture sustainable strategies (CSS). The most influential factors were the choice of R gene (accounting for 40% of the variance) and epidemic intensity before R deployment (30%). The connectivity of the landscape alone accounted for 8% of the variance.

**Figure 4:**
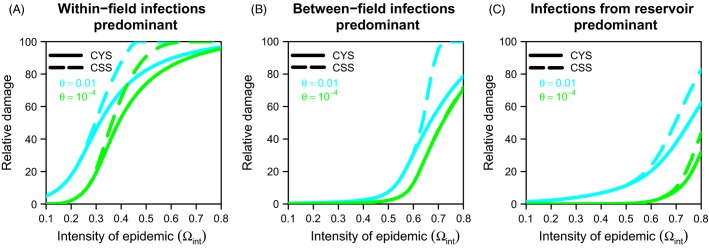
Comparison of the damage reduction achieved with constant-mixture yield strategies and constant-mixture sustainable strategies in three contrasting patterns of landscape connectivity. (A) Effects of epidemic intensity before R deployment (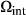) on the relative damage obtained with constant-mixture yield strategies (CYS) and constant-mixture sustainable strategies (CSS) in a landscape in which 90% of the infections are within-field infections 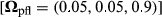. The range of *θ* illustrates a resistance gene typically requiring 1 nucleotide substitution in the avirulence gene of the pathogen to be broken down. (B) As for (A) but for a landscape in which 90% of infections are between-field infections 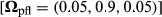. (C) As for (A) but for a landscape in which 90% of the infections are infections from the reservoir 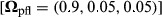.

The yield performance of CSS was compared to that of CYS in Fig. 4 for a R gene typically requiring one mutation to be broken down. It defined thresholds for epidemic intensity below which relative damages were identical for both strategies (i.e., the solid and dashed lines merge in Fig. 4). These thresholds were low (epidemic intensities <0.25) for landscapes in which within-field infections predominated (Fig. 4A), but much higher (>0.55) elsewhere (Fig. 4B,C). In situations in which within-field infections predominated, relative damages for CSS were always 100% for epidemic intensities >0.6 (Fig. 4A). It is therefore not possible to use the R gene and to ensure that the mean proportion of infected R plants is below 5% in all fields and all years. On average, the additional relative cost incurred when using a CSS rather than a CYS was 8% in landscapes in which within-field infections prevailed and 3% when the other two routes of infection predominated. The use of variable-mixture sustainable strategies (VSS) systematically improved performance in landscapes in which (i) within-field infections prevailed (the mean additional relative cost decreasing from 8% to 4%) and (ii) infections originating from the reservoir prevailed (the mean additional relative cost of 3% being converted into a mean additional relative benefit of 1%). However, VSS did not decrease the range of epidemic intensities in situations in which the adoption of CSS was impossible (not illustrated).

For R genes typically requiring two mutations to be broken down, the picture was much simpler (Figure S4). CSS substantially increased the damage due to the pathogen when the dynamics of the epidemic were driven principally by within-field infections (8% on average) (Figure S4A). No such losses occurred with the other two patterns of landscape connectivity (Figure S4B, C).

## Discussion

We analyzed a model explicitly linking the epidemiological and population genetics processes occurring at nested levels of biological organization: (i) within-host population genetics and evolutionary processes and (ii) between-host epidemiological processes. If we assume that the initial pathogen population has never been exposed to a resistant cultivar, then the model describes, at the landscape scale, the dynamics of emergence of a resistance-breaking pathogen and the associated epidemiological dynamics during a succession of cropping seasons in which the resistant cultivar can be sown in variable proportions. We investigated the factors determining the levels of disease control obtained with deployment strategies using the principles of cultivar mixture only (constant-mixture strategies) or using cultivar mixture and rotation (variable-mixture strategies). Two management alternatives were also considered, only minimizing pathogen damage in the short term (yield strategies) or, following Gilligan (2008), reconciling the conflicting goals of reducing damage in the short term while preserving resistance durability in the longer term (sustainable strategies).

In our simulations, deployment of the R cultivar controlled epidemics efficiently. Assuming that the range of parameter variations explored (Table1) is representative of production situations worldwide, CSS reduced damage by more than 90% in 65% of them. Interestingly, R genes conferring resistance to viruses were found to be durable in 68% of the deployment ‘stories’ complied worldwide by García-Arenal and McDonald (2003). The choice of R gene was found to be a key factor for effective disease control. The dedicated parameter *θ* can be estimated *in planta* with high-throughput sequencing techniques when the mutations breaking down the resistance are known (Fabre et al. 2012b). This is now the case for many viral pathosystems (Kang et al. 2005; Moury et al. 2011). Resistance-breaking mutants, now easily obtained in laboratory conditions, are representative of the resistance-breaking isolates found in field conditions (e.g., Moury et al. 2004; Ayme et al. 2006; Hajimorad et al. 2011).

The effect of landscape on plant–pathogen evolution remains unclear, in both cultivated (Mundt 2014) and natural (Meentemeyer et al. 2012) ecosystems. Several studies have considered the management of epidemics at this scale (e.g., Gilligan et al. 2007; Parnell et al. 2009, 2010; Skelsey et al. 2010; Filipe et al. 2012), mostly without taking pathogen evolution into account (but see Papaïx et al. 2013), but only a few have focused specifically on landscape connectivity. Sullivan et al. (2011) found that establishing conservation habitat corridors in fragmented landscapes also increased the incidence of vectorborne plant parasites. Parnell et al. (2009, 2010) found that the dynamics of Asiatic citrus canker and the optimal strategies for its eradication depended on the spatial configuration of its host in the landscape. Skelsey et al. (2010) compared the effect of S and partially R potato cultivar mixtures at three levels on potato late blight epidemics. For their specific pathosystem, within-field mixtures provided the best control. Papaïx et al. (2014) investigated, in a more general context, but without considering pathogen evolution, how the basic components of landscape functional connectivity (proportion and aggregation of S and R cultivars, pathogen fitness on these cultivars, dispersal ability) affected epidemics for an airborne foliar disease. They demonstrated major effects of (i) the proportion of fields sown with a qualitative R cultivar and (ii) their aggregation within the landscape. In our model, the parameter 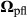 defines functional connectivity in a landscape containing only the S cultivar before R deployment. It results from interactions between the physical characteristics of the host habitats (except for host density, see below) and the dispersal capacities of the pathogen (Meentemeyer et al. 2012). This parameter has a major impact on the dynamics of R breakdown. Over the range of production situations explored (Table1), CYS reduced the damage by more than 90% in 32% of situations in which within-field infection was the prevailing route of infection, 59% of situations in which between-field infection predominated and 68% of situations in which infections originating from the reservoir prevailed. In our setting, overall host density in the area determined epidemic intensity before resistance deployment (parameter 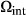). As previously reported (e.g. Skelsey et al. 2010, 2013), reducing the crop acreage and the size of the wild host habitat decreases 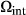 and leads to the efficient control of epidemics.

The efficiency of R deployment strategies is favored by frequent between-field infections. This is because the numerous between-field infection events occurring in these landscapes include some in which a nonadapted pathogen from a field of susceptible plants is introduced into a field of resistant plants, resulting in unsuccessful infection. These failed infections are particularly probable if (i) the counter-selection of resistance-breaking pathogens in the S cultivar is strong and (ii) the proportions of S and R fields are close to 0.5. This effect is known as the dilution effect (Mundt 2002). In practice, the frequency of between-field infections relative to within-field infections can be increased by designing landscapes with, for a given area, a larger number of smaller (genetically homogeneous) fields (i.e. increasing the number of plant genotype units and decreasing the plant genotype unit area). Using an epidemiological model (without pathogen evolution), Mundt and Brophy (1988) showed that, for a given overall host area, increasing the number of genotype units increases the effectiveness of cultivar mixtures and, consequently, disease control. For given field number and size, between-field infections are also favored by random, rather than aggregated, patterns of S and R cultivars conferring complete resistance (Papaïx et al. 2014).

Yield and sustainable strategies were the most effective in landscapes in which infection from the reservoir was the prevailing route of infection. This situation can be described as pathogen spillover (Daszak et al. 2000). The gain was found to be greater for higher epidemic intensities than for landscapes in which between-field infections prevailed (Figs2 vs 3 for 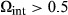). In the model, the pathogen load of the reservoir drove the rate of infection of crops from this compartment and was itself dependent on the intensity of epidemics on crops (eqn 2). Deploying a R cultivar initially reduces the intensity of the epidemic through a dilution effect, which, in turn, reduces the pathogen load of the reservoir. This ‘serial inoculum dilution’ mechanism is effective provided that the frequency of the resistance-breaking pathogen variant in the reservoir remains low (i.e. as long as only few R plants are infected). This mechanism is also effective because we assumed an initial low frequency of the resistance-breaking variant in the reservoir, equal to the mutation-selection balance. This is likely if the R gene is newly introduced from far-off environment but also if the R gene exists in the local wild hosts at low frequency, a condition favored by a high genetic diversity of wild hosts. In practice, the relative proportion of infections originating from the reservoir could be increased by manipulating the wild host community, as shown by Power and Mitchell (2004) for a virus of annual wild and cultivated grasses. Theoretically, it would be of interest to determine which functional connectivity parameters optimize disease control. The existence of an optimum is suggested by the peaking of pathogen dispersal between habitat patches at intermediate scales of habitat heterogeneity relative to the dispersal capacity of pathogens (Skelsey et al. 2013).

One of our key findings concerns variable-mixture strategies. Resistance management strategies to date have focused on the use of the same mixture every year (van den Bosch and Gilligan 2003; Ohtsuki and Sasaki 2006; Sapoukhina et al. 2009; Skelsey et al. 2010; Fabre et al. 2012a; Bourget et al. 2013; Lo Iacono et al. 2013; Papaïx et al. 2013). Such strategies are inevitable for perennial crops (Sapoukhina et al. 2009), but temporal shifts in the mixture of S and R fields are possible for annual crops. Variable-mixture strategies provided a substantial additional benefit over constant-mixture strategies (i) when within-field infections predominated, regardless of the gene introduced (Fig. 1B,C), and (ii) when infections from the reservoir predominated, for R genes typically requiring only one mutation to be broken down (Fig. 3B). By contrast, variable-mixture strategies were useless when between-field infections predominated (Fig. 2B,C) as the dilution effect is maximal when the proportions of S and R fields are similar. Variable-mixture strategies can also be used to circumvent the conflict between efficient epidemic control and R durability. Most optimal variable-mixture strategies combined the principles of cultivar mixture (during a season) and mixture alternation (i.e. between seasons). The best strategies for managing insecticide (Mani 1989) and antibiotic (Masterton 2010) resistances also involved maximizing treatment heterogeneity (REX consortium, 2013), by varying them in time and space. Almost nearly 20% of optimal variable-mixture strategies involve switching the proportion of R fields from one extreme to the other. Such periodic strategies are particularly interesting in terms of their acceptability to farmers as they are similar to crop rotations, a popular agronomic practice worldwide.

The added value of linking within- and between-host dynamics in nested models for studies of disease emergence is recognized (van den Bosch and Gilligan 2003; Mideo et al. 2008). In our model, a single parameter summarizes the within-host dynamics: the mutation-selection balance between nonadapted and resistance-breaking pathogen variants in S hosts. Despite this simplicity, interactions between within-host and between-host processes accounted for 8% and 13% of the relative damage obtained with CYS and CSS, respectively (Figure S2). There was an interesting interaction between landscape connectivity and the choice of R gene, which determined the mutation-selection balance. R genes typically requiring two mutations, rather than just one, are particularly effective when infections originating from the reservoir predominate (Fig. 3B vs C). Careful R gene choice was less crucial elsewhere (Figs1 and 2 panels B and C). It follows that associating R gene choice with control methods (either chemical, cultural, or biological) decreasing epidemic intensity and dedicated landscape planning could be the corner stone to achieve integrated and sustainable disease management.

Several hypotheses may have important effects on model outputs. We assumed that the fitness effects of mutations are constant, implying that resistance-breaking mutants revert to their initial frequencies after removal of the R gene. This has been observed (or inferred) for some viruses (e.g., ToMV – *Tm1* gene, Harrison 2002; PVY – *Pvr4* gene, Janzac et al. 2010; tobamovirus – *L* gene, Fraile et al. 2011), but is not general rule for plant–pathogen (Torres-Barcelo et al. 2010; Mundt 2014). Like most models dealing with the evolution of resistance to xenobiotics (REX consortium, 2010), we ignored genetic drift. Within plants, bottlenecks occur at many steps of the virus cycle (Fabre et al. 2012b; Gutiérrez et al. 2012). Between plants, narrow bottlenecks occur during transmission (Moury et al. 2007; Betancourt et al. 2008), overwintering (Kiyosawa 1989) or due to environmental stochasticity (Lo Iacono et al. 2013). These bottlenecks may contribute to delay the emergence of resistance-breaking variants. At a larger scale, we assumed that the relative frequencies of the nonadapted and resistance-breaking variants arising from crops remained unchanged in the reservoir compartment. This is clearly a baseline hypothesis, but little evidence is available to affirm or refute it. Natural populations of wild host plants are highly patchy, with many diverse genotypes of the same species, and different environmental conditions between populations (Zhan et al. 2015). Wild hosts are also the main source of disease resistance in crops, and they contain a large diversity of R genes and alleles. All these factors make strong directional selection of one particular pathogen variant over another unlikely. More generally, this point highlights the need for further research at the agro-ecological interface and setting up experiments at this scale.

## Supporting Information

**Figure S1.** Validation of Nelder–Mead optimization for deriving variable-mixture yield strategies and variable-mixture sustainable strategies. **Figure S2.** Sensitivity indices of the relative damage obtained with constant-mixture strategies. **Figure S3.** Hierarchical clustering of the proportion of R fields to be sown for variable-mixture yield strategies in a landscape in which infections from the reservoir predominate. **Figure S4.** Comparison of the damage reduction achieved with constant-mixture yield strategies and constant-mixture sustainable strategies for a resistance gene requiring two mutations to be broken down. **Appendix S1.** Derivation of the optimal variable-mixture strategies with a Nelder–Mead algorithm. **Appendix S2.** Implementation of model analyses. **Table S1.** Correspondence between the epidemic rate parameters (*α*_E_,*β*_C_,*β*_F_) and the parameters (Ω_int_, **Ω**_pfl_). **Table S2.** Additional relative benefit obtained with variable-mixture yield strategies.
