# Supplementary material for: Epidemiological and evolutionary management of plant resistance: optimizing the deployment of cultivar mixtures in time and space in agricultural landscapes
Source: Evol Appl. 2015 Oct 23;8(10):919–32. doi: 10.1111/eva.12304 (PMC4662345; doi:10.1111/eva.12304)
Supplement: Supplementary file 1 [file eva0008-0919-sd1.pdf]

Epidemiological and evolutionary management of plant resistance: optimizing the deployment of cultivar mixtures in time and space in agricultural landscapes.

Supporting Information

Additional supporting information may be found in the online version of this article.

Figure S1. Validation of Nelder-Mead optimization for deriving variable-mixture yield strategies and variable-mixture sustainable strategies.

Figure S2. Sensitivity indices of the relative damage obtained with constant-mixture strategies.

Figure S3. Hierarchical clustering of the proportion of R fields to be sown for variable-mixture yield strategies in a landscape in which infections from the reservoir predominate.

Figure S4. Comparison of the damage reduction achieved with constant-mixture yield strategies and constant-mixture sustainable strategies for a resistance gene requiring 2 mutations to be broken down.

Appendix S1. Derivation of the optimal variable-mixture strategies with a Nelder-Mead algorithm.

Appendix S2. Implementation of model analyses.

Table S1. Correspondence between the epidemic rate parameters $\left(\alpha_E, \beta_C, \beta_F\right)$ and the parameters $\left(\Omega_{int}, \mathbf{\Omega}_{pfl}\right)$.

Table S2. Additional relative benefit obtained with variable-mixture yield strategies.

Figure S1. Validation of Nelder-Mead optimization for deriving variable-mixture yield strategies and variable-mixture sustainable strategies. **(A)** Correlation between the relative damage $\Delta\left(\delta,\boldsymbol{\varphi}_{YS}^{V}\right)$ obtained with variable-mixture yield strategies (VYS) as derived with the Nelder-Mead algorithm (x-axis) for 48 combinations of the model parameters and $\Delta\left(\delta,\boldsymbol{\varphi}_{YS}^{V}\right)$ as derived with the "brute force" algorithm (y-axis). **(B)** Same as **(A)** for variable-mixture sustainable strategies (VSS) (*i.e.* with $\Delta\left(\delta,\boldsymbol{\varphi}_{SS}^{V}\right)$). **(C)** Correlation between the elements of the time series $\boldsymbol{\varphi}_{YS}^{V}$ derived with the Nelder-Mead algorithm (x-axis) for 48 combinations of the model parameters (n=48*5=240) and the elements of the time series $\boldsymbol{\varphi}_{YS}^{V}$ derived with the "brute force" algorithm (y-axis). **(D)** Same as **(C)** for VSS (*i.e.* with $\boldsymbol{\varphi}_{SS}^{V}$).

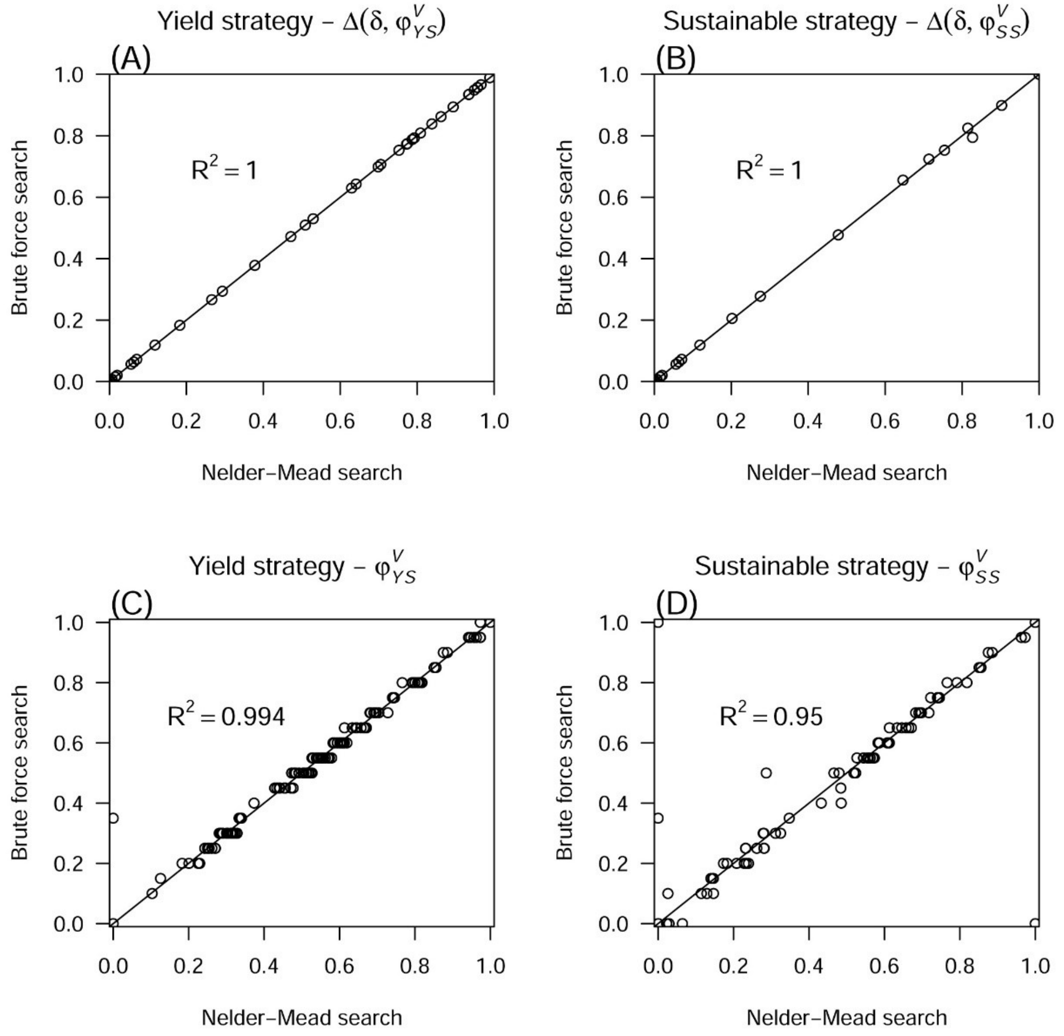

Figure S2. Sensitivity indices of the relative damage obtained with constant-mixture strategies. The factors considered are: (1) θ: characteristics of the resistance gene, (2) λ: characteristics of the reservoir, (3) Ω_{int} : epidemic intensity, (4) $\mathbf{\Omega}_{pfl}$: landscape connectivity and (5) n_y: number of years of resistance deployment. **(A)**: Main effects and most important interactions explaining the relative damage obtained with constant-mixture yield strategies, Δ_{YS}^{C}. **(B)**: Main effects and most important interactions explaining the relative damage obtained with constant-mixture sustainable strategies Δ_{SS}^{C}. The black parts of bars correspond to main effects (effects of the factor alone) and full bars (black, shaded and white parts) correspond to total indices.

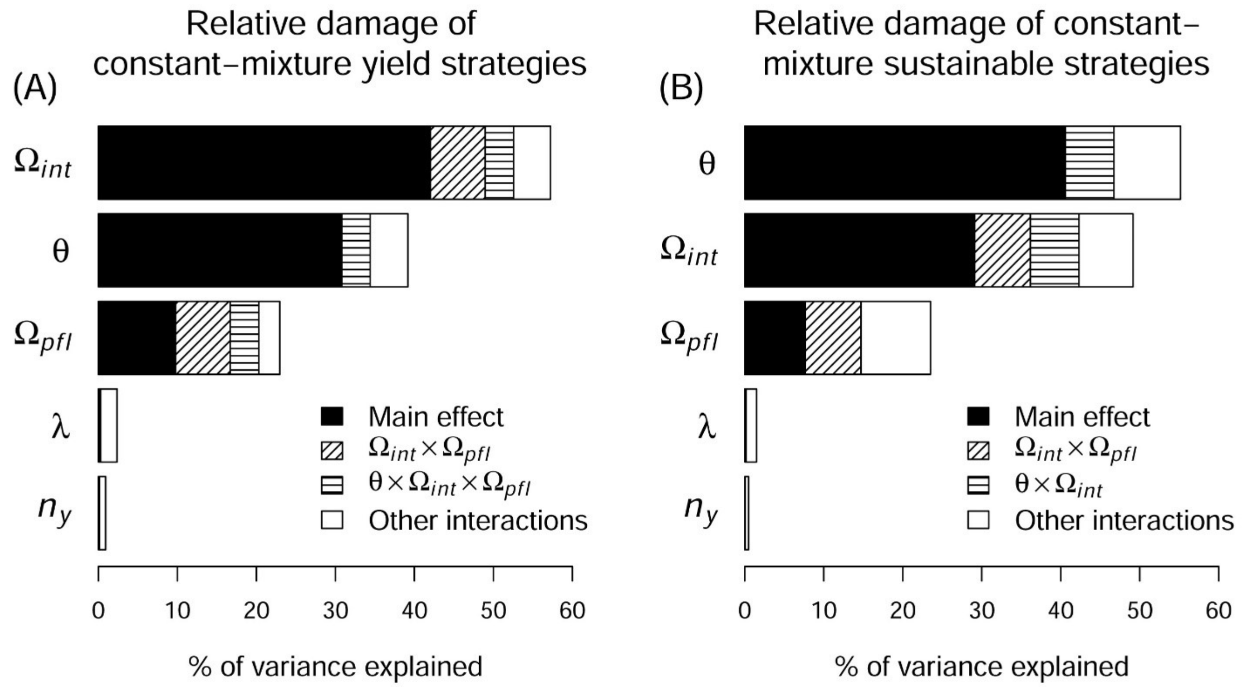

Figure S3. Hierarchical clustering of the proportion of R fields to be sown for variable-mixture yield strategies in a landscape in which infections from the reservoir predominate. (A, B). Clustering in 2 groups of the time series of the optimal proportion of R fields of variable-mixture yield strategies (VYS) when the relative damage obtained with constant-mixture yield strategies (CYS) are < 10% (51 cases out of the 60 parameter combinations displayed in Figure 3, panels B and C). Bars show the mean (+ standard deviation) of the proportion between years 1-3, 4-6, 7-9, 10-12 and 13-15 and the proportion to use with the constant-mixture yield strategies (from years 1 to 15). (C, D) Same as (A, B) when the relative damage obtained with constant-mixture yield strategies are ≥ 10% (9 cases out of 60 analysed).

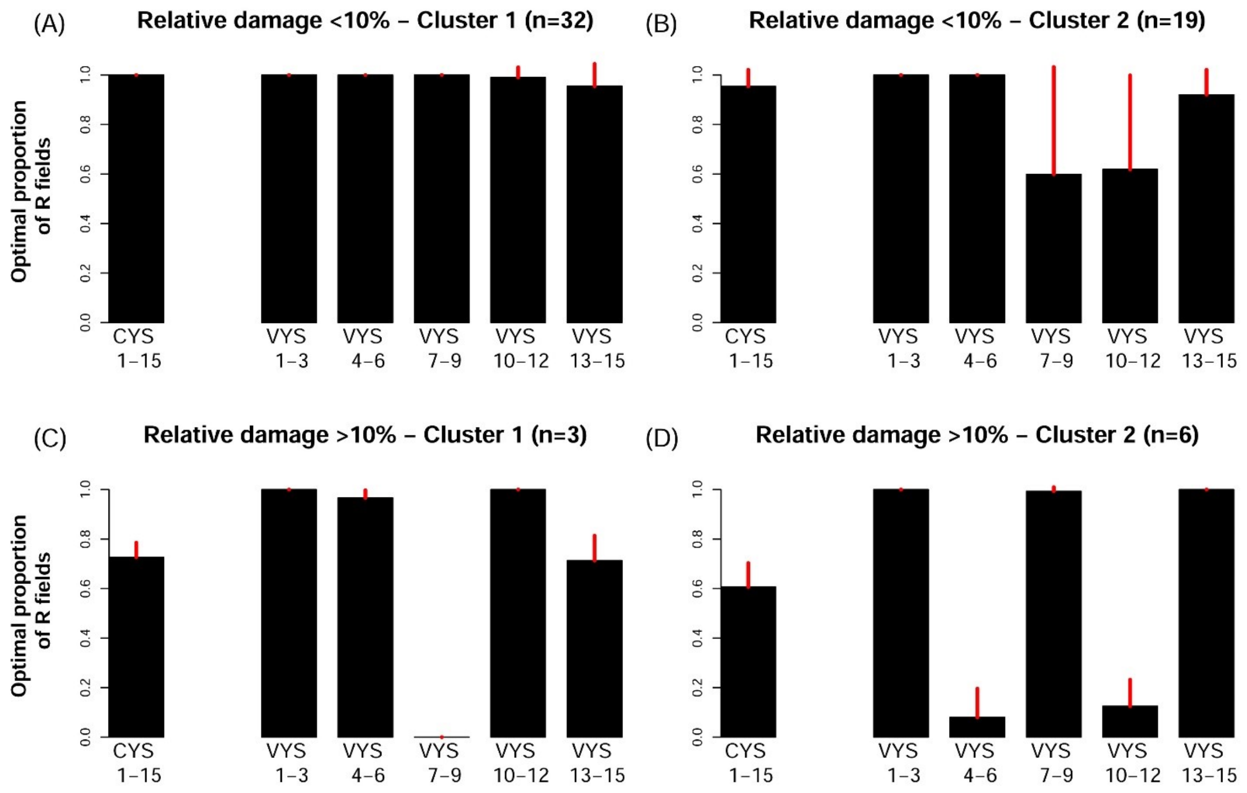

Figure S4. Comparison of the damage reduction achieved with constant-mixture yield strategies (CYS) and constant-mixture sustainable strategies (CSS) for a resistance gene requiring 2 mutations to be broken down. (A) Effects of the epidemic intensity before the deployment of the resistant cultivar (parameter Ω_{int}) on the relative damage obtained with CYS and CSS in a landscape in which 90% of the infections are within-field infections $\left(\boldsymbol{\Omega}_{pfl}=\left(0.05, 0.05, 0.9\right)\right)$. The range of the parameter θ illustrated the case of a resistance gene typically requiring the accumulation of 2 nucleotide substitutions in the avirulence gene of the pathogen to be broken down. (B) Same as (A) in a landscape in which 90% of the infections are between-field infections $\left(\boldsymbol{\Omega}_{pfl}=\left(0.05, 0.9, 0.05\right)\right)$. (C) Same as (A) in a landscape in which 90% of the infections are infections from the reservoir $\left(\boldsymbol{\Omega}_{pfl}=\left(0.9, 0.05, 0.05\right)\right)$.

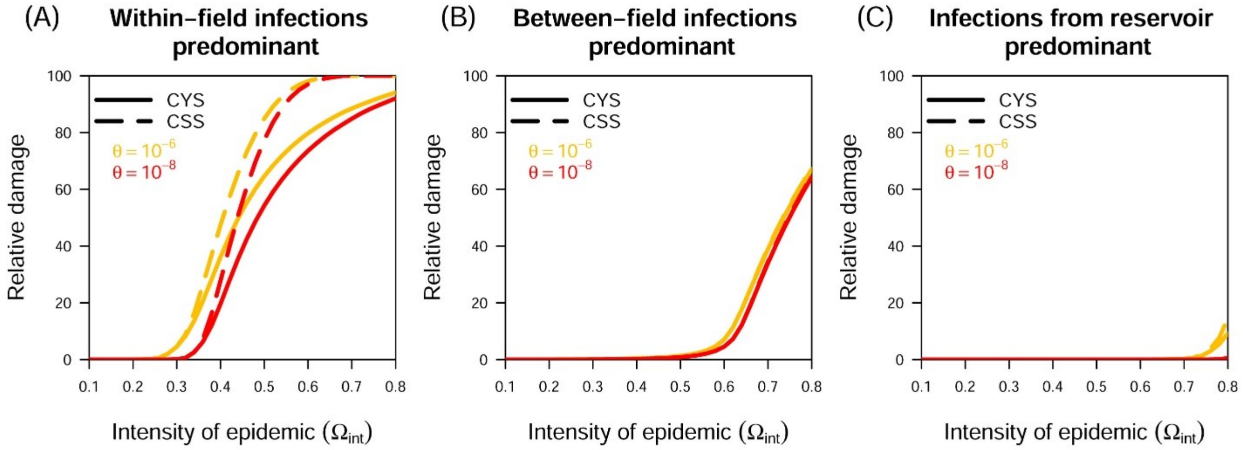

Table S1. Correspondence between the epidemic rate parameters $(\alpha_E, \beta_C, \beta_F)$ and the parameters $(\Omega_{int}, \mathbf{\Omega}_{pfl})$. In a landscape with only susceptible fields, Ω_{int} measures epidemic intensity by the mean proportion of susceptible plants infected in a field during a cropping season. $\mathbf{\Omega}_{pfl} = (\Omega^1_{pfl}, \Omega^2_{pfl}, 1-\Omega^1_{pfl}-\Omega^2_{pfl})$ defines the connectivity between the elements of the landscape (fields, reservoir). Ω^1_{pfl} measures the proportion of infection events originating from the reservoir, Ω^2_{pfl} the proportion of between-fields infection events and the remaining part the proportion of within-field infection events. The correspondences between the two sets of parameters are given for $n_f = 400$ fields, $n_p = 10^4$ plants and $n_d = 120$ days and for all the 60 combinations of Ω_{int} and $\mathbf{\Omega}_{pfl}$ used in the article.

Table S2. Additional relative benefit obtained with variable-mixture yield strategies

**Pathogen renewal in the reservoir**[a]	**Prevailing infection route**[b]	**Additional relative benefit (%)** [c]			
		**min**	**moy**	**max**	***n***
Slow	Within-field	0	0.3	0.5	34
	Between fields	0.03	0.1	0.23	20
	From the reservoir	0	0	0	2
Intermediate	Within-field	1	8	18	43
	Between fields	0.1	0.28	0.46	19
	From the reservoir	5	11	16.2	9
Fast	Within-field	2	17.3	44	46
	Between fields	0.06	0.5	0.9	19
	From the reservoir	7.1	19.2	41.5	11

[a] The rate of renewal of the pathogen load in the reservoir is set by the parameter λ and depends on host lifespans and the relative sizes of the cultivated and reservoir compartments. Slow rates correspond to pathogen populations having a half-life of six years (λ=0.1), intermediate rates to a pathogen population half-life of one year (λ=0.5) and fast rates to a pathogen population half-life of six months (λ=0.9).

[b] The prevailing infection route correspond to landscapes in which, before R gene deployment: (i) 90% of the infection were within-field ($\mathbf{\Omega}_{pfl} = (0.05, 0.05, 0.9)$), (ii) 90% of the infections were between-fields ($\mathbf{\Omega}_{pfl} = (0.05, 0.9, 0.05)$) and (iii) 90% of the infections originated from the reservoir ($\mathbf{\Omega}_{pfl} = (0.9, 0.05, 0.05)$).

[c] The minimum, mean and maximum of the additional relative benefit obtained with variable-mixture yield strategies (A_{YS}^{V}) are estimated for cases in which the relative damage obtained with constant-mixture yield strategies exceeded 10% ($\Delta_{YS}^{C} \geq 10\%$, n cases of the 60 parameter combinations explored for each λ and $\mathbf{\Omega}_{pfl}$).

Appendix S1. Derivation of the optimal variable-mixture strategies with a Nelder-Mead algorithm.

In this note we provide evidence that a Nelder-Mead optimization algorithm efficiently identifies variable-mixture strategies of resistance deployment. We only considered variable strategies lasting $n_y = 15$ years where the proportion of R fields sown in the landscape change (possibly) every 3 years.

Deriving the optimal Variable-mixture Yield Strategies (VYS), consists in determining the vector of 5 proportions of R fields $\boldsymbol{\varphi}_{YS}^{V} = \left\{\varphi_{YS}^{1}, \varphi_{YS}^{1}, \varphi_{YS}^{1}, ..., \varphi_{YS}^{5}, \varphi_{YS}^{5}, \varphi_{YS}^{5}\right\}$ minimizing pathogen damage $D(\delta, \boldsymbol{\varphi})$, each proportion being applied during three successive cropping seasons. Similarly, deriving the optimal Variable-mixture Sustainable Strategies (VSS), consists in determining the vector of 5 proportions of R fields $\boldsymbol{\varphi}_{SS}^{V} = \left\{\varphi_{SS}^{1}, \varphi_{SS}^{1}, \varphi_{SS}^{1}, ..., \varphi_{SS}^{5}, \varphi_{SS}^{5}, \varphi_{SS}^{5}\right\}$ minimising damage $D(\delta, \boldsymbol{\varphi})$ while ensuring that the mean proportion of R plants infected never exceed a threshold υ_P preset to 0.05, in no field and during no year. This latter condition is satisfied if the variable $P(\delta, \boldsymbol{\varphi}) = \frac{1}{n_y} \sum_{y \in \Omega_y} \left(\frac{\overline{p}_{R,y}}{\upsilon_P} - 1 \right)$ is null where $\overline{p}_{R,y} = \frac{1}{n_d} \sum_{t=1}^{n_d} \frac{I_{R,y}}{n_p}$ and Ω_y is the set of years where $\overline{p}_{R,y} \geq \upsilon_P$.

For 48 combinations of the main parameters of interest, we checked that a "brute force" search algorithm, testing all possible variable strategies with a 5% step, and a Nelder-Mead minimization algorithm identify close strategies. For the Nelder-Mead optimization, 4 optimizations starting from 4 initial conditions were realized and the one satisfying better the criteria was selected. The 4 initial values were chosen as the strategies giving the 4 lower criteria over 5000 possible strategies sampled uniformly in [0,1] in a latin hypercube design (routine "improvedLHS" of the "lhs" R package). For VSS, as the Nelder-Mead algorithm solves single-objective optimization problem, we minimise $D(\delta, \boldsymbol{\varphi}) + P(\delta, \boldsymbol{\varphi})$. The 48 combinations of the parameters of interest are obtained from all combinations of 3 levels of Ω_{int} [0.2, 0.5, 0.8], 4 levels of $\boldsymbol{\Omega}_{pfl}$ [(1/3, 1/3, 1/3), (0.05, 0.05, 0.9), (0.05, 0.9, 0.05), (0.9, 0.05, 0.05)] and 4 levels of θ [10^{-8}, 10^{-4}, 10^{-2}, 0.5].

For both VYS and VSS, the efficiency of the Nelder-Mead algorithm was assessed by plotting the correlation between pathogen damages ($D(\delta, \boldsymbol{\varphi}_{YS}^{V})$ or $D(\delta, \boldsymbol{\varphi}_{SS}^{V})$) obtained with both the "brute force" and the Nelder-Mead algorithms. The high correlations obtained (R^2=1; Figure S1 AB) indicate that the Nelder-Mead algorithm correctly identifies strategies leading to damages very

close to the one obtained with the “brute force” search algorithm.

We also check if the deployment strategies (*i.e.* the elements of the series $\boldsymbol{\varphi}_{YS}^{V}$ and $\boldsymbol{\varphi}_{SS}^{V}$) were identical. A good correlation is obtained for both the VYS (R^2=0.99; Figure S1 C) and the VSS (R^2=0.95; Figure S1 D). In 2 cases out of 96, 1 value differed in the strategies. The first case arises for $\Omega_{int} = 0.5$, $\boldsymbol{\Omega}_{pfl} = (0.9, 0.05, 0.05)$ and $\theta = 10^{-4}$. For the VYS, the optimal strategy identified by the “brute force” search algorithm was $\boldsymbol{\varphi}_{YS}^{V} = \{1, 1, 1, 0.35, 1\}$ while it was $\boldsymbol{\varphi}_{YS}^{V} = \{1, 1, 1, 0, 1\}$ with the Nelder-Mead algorithm. Despite this difference between years 10 to 12, the yield performance were close (0.0019 versus 0.0023). The second case arises for $\Omega_{int} = 0.5$, $\boldsymbol{\Omega}_{pfl} = (0.05, 0.05, 0.9)$ and $\theta = 10^{-8}$. For the VSS, the optimal strategy identified by the “brute force” search algorithm was $\boldsymbol{\varphi}_{SS}^{V} = \{1, 0, 1, 0, 0.1\}$ while it was $\boldsymbol{\varphi}_{SS}^{V} = \{1, 0, 0, 1, 0.11\}$ with the Nelder-Mead algorithm. Again the yield performance were however very close (0.477 versus 0.478).

Appendix S2. Implementation of model analyses.

The model and analyses were implemented in R software (http://www.r-project.org/). The model was solved with the "lsoda" function (library "deSolve"). The numerical exploration of the model was performed by combining global sensitivity analysis, one-at-a-time analysis and hierarchical clustering methods. The correspondence between the levels of the parameters $\left(\Omega_{int}, \boldsymbol{\Omega}_{pfl}\right)$ used in the simulations and the epidemic rate parameters $\left(\alpha_E, \beta_C, \beta_F\right)$ is given in Table S1.

Global sensitivity analyses (Saltelli et al. 2008) quantify the relative importance of model parameters by partitioning the variance of output variables into the variance due to the main effects of parameters and the variance due to their interactions. We determined the sensitivity indices of the relative damages obtained with optimal CYS $\left(\Delta_{YS}^{C}\right)$ and with optimal CSS $\left(\Delta_{SS}^{C}\right)$ to the 5 parameters of interest ($\theta, \Omega_{int}, \boldsymbol{\Omega}_{pfl}, \lambda$ and n_y). We first assigned levels of variation accounting for the known biological variabilities $\left(\theta, \Omega_{int}, n_y\right)$ or for a wide range of possible states $\left(\boldsymbol{\Omega}_{pfl}, \lambda\right)$ to each parameter (Table 1). The model was then run for 480 parameter combinations of the corresponding full factorial design. We then estimated sensitivity indices as the proportion of the total variance explained by a given factor alone (main effects) or by its higher order interactions, by fitting analysis of variance (ANOVA) linear models including four-order interactions to the simulations. All parameters were treated as qualitative factors (their levels are detailed in Table 1). The fit of the ANOVA models was very good (>99% of the variance explained), making it possible to determine sensitivity indices accurately. The parameters n_f, n_p and n_d were not included in the analyses, in accordance with the results of the transformation into a dimensionless model described above. Global sensitivity analyses were combined with graphical one-at-a-time analysis of the three most important parameters identified by global sensitivity analyses, to determine the individual impacts of these parameters on the output of the model.

For given values of Ω_{pfl} and λ, the relative damage in CYS and VYS, Δ_{YS}^{C} and Δ_{YS}^{V}, was determined for 60 sets of parameters combining four values of θ (0.01, 10^{-4}, 10^{-6}, 10^{-8}) with 15 values of Ω_{int} (from 0.1 to 0.8 by 0.05). The time series $\boldsymbol{\varphi}_{YS}^{V}$ of VYS corresponding to a combination of parameters for which $\Delta_{YS}^{C} < 10\%$ were classified into two clusters by a hierarchical clustering method (function "hclust", Ward method), after the determination of a Euclidean

distance matrix between the time series (function "dist"). The same was done for the $\boldsymbol{\varphi}_{YS}^{V}$ time series for which $\Delta_{YS}^{C} \geq 10\%$. This threshold of 10% was arbitrarily chosen to distinguish between the optimal CYS for which a substantial margin of improvement exists $\left(\Delta_{YS}^{C} \geq 10\%\right)$. The same analyses were then carried out for sustainable strategies.

Literature cited.

Saltelli, A., M. Ratto, T. Andres, F. Campolongo, J. Cariboni, D. Gatelli, M. Saisana, and S. Tarantola 2008. Global sensitivity analysis: The Primer. Chichester: Wiley. 304 pages.
